# Foley Catheter for Cervical Ripening Across Gestational Ages: A Retrospective Descriptive Study in a Resource-Limited Setting

**DOI:** 10.7759/cureus.110158

**Published:** 2026-06-03

**Authors:** Kawtar Iraqi Houssaini, Mohamed Amine Guennouni, Bouchra Fakhir, Abderraouf Soummani

**Affiliations:** 1 Obstetrics and Gynecology Department, Mohammed VI University Hospital, Marrakesh, MAR; 2 Morphosciences Research Laboratory, Faculty of Medicine and Pharmacy, Cadi Ayyad University, Marrakesh, MAR

**Keywords:** cervical ripening, early gestation, foley catheter, maternal-neonatal outcomes, mechanical induction, resource-limited setting, retrospective descriptive study, term pregnancy

## Abstract

Induction of labor or pregnancy termination is a frequent obstetric intervention when continuation of pregnancy may endanger maternal or fetal health. Mechanical cervical ripening using a Foley catheter has regained clinical interest due to its favorable safety profile, low cost, and ease of use. Compared with pharmacologic agents, it is associated with a lower risk of uterine hyperstimulation while facilitating cervical ripening.

This study aimed to describe the safety, feasibility, and outcomes of Foley catheter use across the three gestational trimesters in a single institution during a period when prostaglandins were temporarily unavailable.

A retrospective descriptive study was conducted at Mohammed VI University Hospital, Marrakesh, Morocco, from October 2024 to February 2025, including 104 patients: 37 in the first trimester (T1), 14 in the second trimester (T2), and 53 in the third trimester (T3). A 16-18-Fr Foley catheter inflated with 50-60 mL saline was used for cervical ripening. In T3, oxytocin infusion was administered after catheter expulsion or removal, whereas in T1 and T2, the catheter was used as the sole induction method. Evaluated endpoints included induction time intervals, success rates, maternal morbidity, and neonatal outcomes.

Among all patients, 72.1% initiated labor within 12 hours of catheter insertion. The mean induction-to-expulsion/delivery interval was 17.5±7.2 hours in T1, 16.8±6.8 hours in T2, and 12.2±5.4 hours in T3. Induction success rates were 100% in T1 and T2 and 94.3% (50/53) in T3. Morbidity was low, with no maternal deaths reported. In T3, among live births, 77.1% of neonates had an Apgar score of ≥8 at five minutes, reflecting an overall favorable neonatal condition in this group.

Foley catheter mechanical induction appears to be a feasible and safe strategy across all gestational trimesters, particularly in resource-limited settings.

## Introduction

Artificial induction of labor is a routine obstetric intervention used when continuation of pregnancy may pose risks to the mother or fetus [1]. Mechanical cervical ripening with a Foley catheter, first described by Embrey and Mollison in 1967 [2], has regained clinical relevance because of its favorable safety profile, low cost, and ease of application [3,4]. Compared with pharmacologic agents, this method is associated with a lower risk of uterine hyperstimulation while facilitating cervical ripening [5].

Most published studies evaluate Foley catheter use in term pregnancies or during mid-trimester termination, frequently in combination with prostaglandins. Evidence supporting its use as a sole induction technique in early gestation remains limited, and to our knowledge, no prior study has reported its use across all three gestational trimesters within a single institutional cohort.

During the study period, the temporary unavailability of prostaglandins at our institution led to the implementation of a fully mechanical induction protocol across all gestational ages. This context provided an opportunity to examine real-world practices and outcomes in the absence of pharmacological alternatives, particularly in a resource-limited setting.

It is important to note that the induction protocol differed across trimesters: in T1 and T2, the Foley catheter was used as the sole induction method without subsequent oxytocin, whereas in T3, oxytocin infusion was initiated after catheter expulsion or removal according to institutional practice. Accordingly, the objective of this study is descriptive, aiming to document trimester-specific clinical outcomes rather than to establish comparative effectiveness. No inferential conclusions are drawn from cross-trimester differences.

This study therefore reports the outcomes of Foley catheter cervical ripening across gestational trimesters and provides real-world data on its feasibility in settings where access to pharmacological agents is limited.

## Materials and methods

Study design and setting

After obtaining approval from the Ethics Committee of the Mohammed VI University Hospital (approval number: 011/2026), a retrospective descriptive study was conducted over a five-month period from October 2024 to February 2025 in the Department of Gynecology and Obstetrics at Mohammed VI University Hospital, Marrakesh, Morocco. Data were extracted from electronic medical records. Due to the temporary unavailability of prostaglandins, an exclusively mechanical induction protocol was implemented across all gestational ages. Results are presented descriptively by trimester, that is, T1 (<14 weeks), T2 (14-27+6 weeks), and T3 (≥28 weeks), with the T3 group in this study restricted to term pregnancies (≥37 weeks).

Eligibility criteria

Inclusion criteria were the following: all pregnant women requiring induction of labor or pregnancy termination during the study period, regardless of gestational age or indication. For term pregnancies (T3), a Bishop score below 6 was required for cervical assessment. It is acknowledged that the Bishop score is validated for term pregnancies and has no established role in early gestation.

Exclusion criteria were as follows: prior cesarean delivery or uterine surgery, placenta previa, unexplained third-trimester bleeding, suspected chorioamnionitis, or active maternal infection. Although recent data suggest mechanical methods may be safe in selected women with prior cesarean delivery [6], our institutional protocol excluded scarred uteri during the study period.

Induction protocol

A 16-18-French Foley catheter was inserted intracervically under aseptic conditions using a speculum, and the balloon was inflated with 50-60 mL of sterile saline [7]. In T1 and T2 (early pregnancy), induction was performed using the Foley catheter as the sole method, without adjunctive prostaglandins or oxytocin, consistent with the literature distinguishing isolated mechanical from combined approaches [8]. In T3 (term pregnancy), oxytocin infusion (5 IU in 500 mL normal saline, starting at 2 mIU/min and increased by 2 mIU/min every 30 minutes until adequate uterine contractions were achieved [9]) was administered following catheter expulsion or removal. Maternal and fetal monitoring was initiated after catheter placement in all cases. A second mechanical attempt was allowed after 24 hours in the absence of cervical progression, and induction was discontinued if infection was suspected.

Time points were defined as follows: T0 (catheter insertion), T1 (catheter expulsion or onset of labor), and T2 (delivery or complete expulsion of gestational contents).

Outcomes

Primary endpoints included catheter-to-expulsion interval (T0-T1), time to onset of regular uterine contractions, and total induction-to-expulsion/delivery interval (T0-T2), reported by trimester.

Secondary endpoints included delivery mode, indications for cesarean section, maternal morbidity (postpartum hemorrhage, retained products of conception, puerperal infection), and the need for a second catheter attempt.

Induction success was defined according to gestational context. In T3, success corresponded to vaginal delivery without cesarean section for failed induction. In T1 and T2, success was defined as complete fetal expulsion via the vaginal route, irrespective of subsequent uterine evacuation for retained products, as trophoblastic retention represents a recognized post-expulsion event rather than induction failure. Success rates were calculated as the proportion of patients achieving these outcomes within each trimester group.

Neonatal outcomes included Apgar score at five minutes and early neonatal mortality (within seven days). Neonatal monitoring followed institutional protocols, including continuous fetal heart rate monitoring. Apgar scores were assessed by the attending neonatologist. In T1 and T2, neonatal assessment was not applicable given the absence of viable neonates in most cases.

Statistical analysis

Quantitative variables are presented as mean±standard deviation (SD) for normally distributed data or median (range) when appropriate. Categorical variables are expressed as frequencies and percentages. Results are reported by trimester and for the overall cohort where relevant.

Statistical analyses were performed using jamovi (version 2.3). Given the descriptive design, no inferential comparisons between groups were conducted.

## Results

Study population and maternal characteristics

A total of 104 patients underwent Foley catheter induction during the study period and were included in the analysis: 37 (35.6%) in T1, 14 (13.5%) in T2, and 53 (51%) in T3. Maternal baseline characteristics are summarized in Table 1. The mean maternal age was 31.7±7.9 years in T1, 29.2±8.1 years in T2, and 28.6±6.9 years in T3. The mean gravidity was 3.1±1.7, 2.2±1.4, and 2.4±1.5 in T1, T2, and T3, respectively.

**Table 1:** Patient characteristics, induction outcomes, and maternal and neonatal results stratified by gestational trimester Values are presented as mean±SD, median (range), or n (%). Hyphen (-) indicates no cases. Time-interval variables were normally distributed and are expressed as mean±SD.

Variable		1st trimester (n=37)	2nd trimester (n=14)	3rd trimester (n=53)
Patient characteristics				
Maternal age (years)	Mean±SD	31.7±7.9	29.2±8.1	28.6±6.9
	Median (range)	31.0 (18-46)	31.0 (18-44)	28.0 (19-42)
Gravidity	Mean±SD	3.1±1.7	2.2±1.4	2.4±1.5
	Median (range)	3.0 (1-7)	2.0 (1-6)	2.0 (1-7)
Parity	Mean±SD	1.7±1.4	1.7±1.3	2.1±1.3
	Median (range)	2.0 (0-5)	1.0 (0-4)	2.0 (0-6)
Indications for induction: n (%)				
Missed abortion		36 (97.3%)	6 (42.9%)	-
Partial molar pregnancy		1 (2.7%)	-	-
Fetal malformations		-	2 (14.3%)	1 (1.9%)
Intrauterine fetal death (IUFD)		-	2 (14.3%)	5 (9.4%)
Medical termination of pregnancy		-	2 (14.3%)	-
Twin pregnancy		-	1 (7.1%)	-
Hypertensive disorder (hypertensive disorders of pregnancy (HTAG)/preeclampsia)		-	1 (7.1%)	5 (9.4%)
Diabetes mellitus (type 1, type 2, or gestational)		-	-	14 (26.4%)
Prelabor rupture of membranes (PROM)		-	-	7 (13.2%)
Post-term pregnancy		-	-	11 (20.8%)
Amniotic fluid abnormality		-	-	6 (11.3%)
Thrombocytopenia		-	-	4 (7.5%)
Induction characteristics				
Catheter-to-expulsion interval (hours): mean±SD		11.4±5.3	10.8±4.9	5.8±3.1
Time to onset of contractions (hours): mean±SD		11.6±5.7	10.9±5.1	7.0±3.4
Induction-to-delivery interval (hours): mean±SD		17.5±7.2	16.8±6.8	12.2±5.4
Labor onset within 12 hours: n (%)		24 (64.9%)	9 (64.3%)	42 (79.2%)
Second catheter attempt required: n (%)		3 (8.1%)	1 (7.1%)	-
Maternal outcomes				
Induction success rate (fetal expulsion or vaginal delivery): n (%)		37 (100%)	14 (100%)	50 (94.3%)
Cesarean section: n (%)		-	-	3 (5.7%)
Failure to progress/dystocia		-	-	2 (3.8%)
Suspected acute fetal distress		-	-	1 (1.9%)
No complications: n (%)		27 (73%)	9 (64.3%)	48 (90.6%)
Infection (chorioamnionitis/endometritis)		2 (5.4%)	3 (21.4%)	4 (7.5%)
Retained products of conception		6 (16.2%)	-	-
Postpartum hemorrhage		2 (5.4%)	2 (14.3%)	1 (1.9%)
Total complications: n (%)		10 (27%)	5 (35.7%)	5 (9.4%)
Neonatal outcomes				
Apgar score of ≥8 at 5 min: n (%)		N/A	N/A	37/48 (77.1%)
Early neonatal deaths (first 7 days): n (%)		N/A	2 (14.3%)	-

Indications for induction

Indications varied by trimester and are detailed in Table 1.

In T1 (n=37), the predominant indication was missed abortion (97.3%; n=36), followed by partial molar pregnancy (2.7%; n=1).

In T2 (n=14), indications included missed abortion (42.9%; n=6), intrauterine fetal death (14.3%; n=2), fetal malformations (14.3%; n=2), and medical termination of pregnancy (14.3%; n=2), both cases related to end-stage renal failure, as well as twin pregnancy (7.1%; n=1) and hypertensive disorder (7.1%; n=1).

In T3 (n=53), indications were heterogeneous: diabetes mellitus (pre-existing or gestational) (26.4%; n=14), post-term pregnancy (20.8%; n=11), prelabor rupture of membranes (13.2%; n=7), amniotic fluid disorders (11.3%; n=6), intrauterine fetal death (9.4%; n=5), hypertensive disorders of pregnancy (9.4%; n=5), thrombocytopenia (7.5%; n=4), and fetal malformations (1.9%; n=1).

Induction characteristics and time intervals

Induction time intervals are presented descriptively in Table 1.

In T1, the mean catheter-to-expulsion interval was 11.4±5.3 hours, the mean time to onset of contractions was 11.6±5.7 hours, and the mean induction-to-expulsion interval was 17.5±7.2 hours. Labor was initiated within 12 hours in 64.9% of patients (n=24).

In T2, corresponding intervals were 10.8±4.9, 10.9±5.1, and 16.8±6.8 hours, respectively, with 64.3% (n=9) initiating labor within 12 hours.

In T3, following catheter expulsion or removal and the initiation of oxytocin infusion, the mean catheter-to-expulsion interval was 5.8±3.1 hours, the time to onset of contractions was 7.0±3.4 hours, and the induction-to-delivery interval was 12.2±5.4 hours. Labor was initiated within 12 hours in 79.2% of patients (n=42).

Across the entire cohort, 72.1% of patients initiated labor within 12 hours of catheter insertion.

A second catheter attempt was required in three patients (8.1%) in T1 and one patient (7.1%) in T2, whereas none was required in T3. For these cases, time intervals were calculated from the second catheter insertion.

Maternal outcomes

All patients in T1 and T2 achieved vaginal fetal expulsion (100%). In T3, 50 patients (94.3%) delivered vaginally, and three (5.7%) underwent cesarean section: two for failure to progress and one for suspected acute fetal distress (Table 1). Infectious morbidity (chorioamnionitis or endometritis) occurred in two T1 patients (5.4%), three T2 patients (21.4%), and four T3 patients (7.5%), all successfully managed with antibiotic therapy. Postpartum hemorrhage was reported in two patients (5.4%) in T1, two (14.3%) in T2, and one (1.9%) in T3; all cases were managed conservatively with uterotonic agents, without the need for transfusion or surgical intervention. Retained products of conception occurred in six patients (16.2%) in T1, an expected outcome in early gestation, and in none of the T2 or T3 cases. All were managed by surgical uterine evacuation. These cases were not considered induction failures. No maternal deaths were recorded.

Neonatal outcomes

Neonatal outcomes are presented in Table 1. In T3, among live births (excluding five cases of intrauterine fetal death; n=48), 37 neonates (77.1%) had an Apgar score of ≥8 at five minutes. No early neonatal deaths were recorded in T1 or T3. In T2, two early neonatal deaths were recorded (14.3%), both occurring in the context of severe underlying fetal pathology. The overall early neonatal fatality rate for the entire cohort was 1.07% (2/104).

## Discussion

Cervical ripening refers to the progressive softening, shortening, effacement, and dilatation of the cervix. The common methods used to promote cervical ripening and labor induction include pharmacological agents (such as oxytocin and prostaglandins E1 and E2) and mechanical approaches (such as balloon catheters and hygroscopic dilators). A historical review by Patabendige et al. highlights the renewed interest in mechanical methods, particularly the transcervical Foley catheter, following decades of pharmacological predominance [5].

Recent meta-analyses suggest that Foley catheter use is associated with outcomes comparable to prostaglandins for labor induction while reducing the risk of uterine tachysystole and adverse perinatal events. Optimal parameters typically include a single-balloon catheter inflated with 30-40 mL without traction. Current evidence supports the use of mechanical methods as a reliable first-line or complementary option for labor induction, particularly within a safety-oriented and individualized obstetric approach [10].

According to the World Health Organization, 20-30% of deliveries are induced [11]. Induction methods are selected based on obstetric assessment, including the Bishop score [12]. Mechanical methods, particularly the Foley catheter, are associated with lower cesarean rates and fewer maternal complications compared with pharmacological agents, which carry a higher risk of uterine hyperstimulation and rupture [13]. Their low cost, reproducibility, and minimal systemic effects make them especially suitable for low-resource settings [14].

Our findings suggest that Foley catheter use was associated with acceptable outcomes and a low complication rate across gestational groups. As expected, indications for induction differed markedly according to gestational age. In T1, missed abortion accounted for nearly all cases (97.3%; n=36/37). In T2, indications were more heterogeneous, with missed abortion remaining the most frequent indication (42.9%; n=6/14), followed by intrauterine fetal death, fetal malformations, and medical termination of pregnancy, each accounting for 14.3% of cases. In T3, the leading indications were gestational or pre-existing diabetes mellitus (26.4%; n=14/53), post-term pregnancy (20.8%; n=11/53), and prelabor rupture of membranes (13.2%; n=7/53). Similar distributions have been reported by Gonsalves et al., who identified post-term pregnancy (26.5%), growth restriction (27.9%), and gestational diabetes (17.6%) as major indications for induction [15]. Comparable findings were also described by Patabendige and Jayawardane [16].

Overall, 72.1% of patients in the study population initiated labor within 12 hours of catheter insertion, with higher rates observed in term pregnancies (79.2%) than in T1 (64.9%) or T2 (64.3%). Larger balloon volumes have been associated with improved cervical dilation without significantly affecting cesarean section rates [1,17]. Cromi et al. [18] also observed that limiting cervical maturation to approximately 12 hours may help shorten induction-to-delivery intervals. Similar findings were reported by Rossard et al. [19] and Lamourdedieu et al. [20], who observed that mechanical stimulation alone can trigger uterine activity in 25.6% of cases. The effect of the Foley catheter is thought to involve cervical stretching, membrane separation, and endogenous prostaglandin release, thereby facilitating progression from the latent to the active phase, as described by Gu et al. [1] and Tuuli et al. [21].

Enhancing Foley catheter induction with adjunctive agents such as oxytocin or misoprostol may improve labor outcomes. In our protocol, oxytocin was routinely administered following catheter expulsion in T3. Lee et al. and Kruit et al. reported higher vaginal delivery rates and lower rates of perinatal asphyxia and immediate postpartum hemorrhage with combined approaches [22,23]. Similarly, Liu et al. [9] found that simultaneous Foley-oxytocin induction increased delivery within 24 hours in nulliparous women without increasing cesarean or adverse outcome rates. Collectively, these observations suggest that differences across gestational stages are more likely related to variations in cervical physiology and clinical context than to intrinsic differences in Foley catheter performance.

The cesarean section rate in our cohort (5.7%) was lower than those reported by Rossard et al. (35.9%) [19], Pez et al. (22%) [14], and Patabendige and Jayawardane (14%) [16]. However, this likely reflects selection bias related to the exclusion of women with prior uterine scars, suspected macrosomia, and other high-risk conditions, limiting comparison with broader obstetric populations. Prior studies similarly found no significant difference in cesarean rates between Foley catheter and dinoprostone or Cook double-balloon catheters [8,24]. A recent Chinese randomized trial reported comparable vaginal birth rates, with dinoprostone associated with more uterine hyperkinesia and neonatal asphyxia and Foley with higher maternal infection risk [25]. Combination strategies further enhance efficacy without compromising safety, as shown in a recent review [26].

In our unit, Foley catheter use remained a cost-effective and efficient approach, with an 80.8% success rate and no major complications, consistent with findings reported by Rossard et al. [19] and Lamourdedieu et al. [20], although infectious complications such as chorioamnionitis have been described by other authors [14,27]. As the Foley catheter was used alone in early gestation due to misoprostol unavailability, the shorter induction intervals observed at term likely reflect both more favorable cervical physiology and the use of adjunctive oxytocin.

Study limitations

This retrospective single-center study may be subject to selection bias and impaired generalizability due to exclusion criteria. Differences in induction protocols and trimester-specific definitions of success limited group comparisons. The Bishop score is less applicable in early gestation, and clinical heterogeneity may have affected outcomes. Neonatal evaluation was limited, and the small number of second-trimester cases restricted subgroup analysis.

## Conclusions

This retrospective descriptive study suggests that Foley catheter mechanical induction is feasible across different stages of pregnancy in a resource-limited setting, with a low rate of major complications. Induction success, defined according to trimester-specific clinical objectives, was achieved in all T1 and T2 patients and in 94.3% (50/53) of T3 patients. These findings support the use of this method as a practical option for cervical ripening and induction when pharmacological agents are unavailable or contraindicated. Further prospective and multicenter studies with standardized protocols and more comprehensive neonatal assessment would help clarify its role in clinical practice.
